# 3D-printed electrochemical glucose device with integrated Fe(II)-MOF nanozyme

**DOI:** 10.1007/s00604-023-05860-6

**Published:** 2023-06-24

**Authors:** Eleni Koukouviti, Alexios K. Plessas, Varvara Pagkali, Anastasios Economou, Giannis S. Papaefstathiou, Christos Kokkinos

**Affiliations:** 1 Laboratory of Analytical Chemistry, Department of Chemistry, National and Kapodistrian University of Athens, 15771 Athens, Greece; 2 Laboratory of Inorganic Chemistry, Department of Chemistry, National and Kapodistrian University of Athens, 15771 Athens, Greece

**Keywords:** Glucose, MOF, 3D printing, Sensor, Enzymatic-free, Voltammetry, Nanozyme

## Abstract

Estimation of glucose (GLU) levels in the human organism is very important in the diagnosis and monitoring of diabetes. Scientific advances in nanomaterials have led to the construction of new generations of enzymatic-free GLU sensors. In this work, an innovative 3D-printed device modified with a water-stable and non-toxic metal–organic framework of iron (Fe(II)-MOF), which serves as a nanozyme, has been developed for the voltammetric determination of GLU in artificial sweat. In contrast to existing MOF-based GLU sensors which exhibit electrocatalytic activity for the oxidation of GLU in alkaline media, the nanozyme Fe(II)-MOF/3D-printed device can operate in the acidic epidermal sweat environment. The enzymatic-free GLU sensor is composed of a 3-electrode 3D-printed device with the MOF nanozyme immobilized on the surface of the working electrode. GLU sensing is conducted by differential pulse voltammetry without interference from other co-existing metabolites in artificial sweat. The response is based on the oxidation of glucose to gluconolactone, induced by the redox activity of the Fe-centers of the MOF. GLU gives rise to an easily detectable and well-defined voltammetric peak at about − 1.2 V and the limit of detection is 17.6 μmol L^-1^. The synergy of a nanozyme with 3D printing technology results in an advanced, sensitive, and low-cost sensor, paving the way for on-skin applications.

**Graphical abstract:**

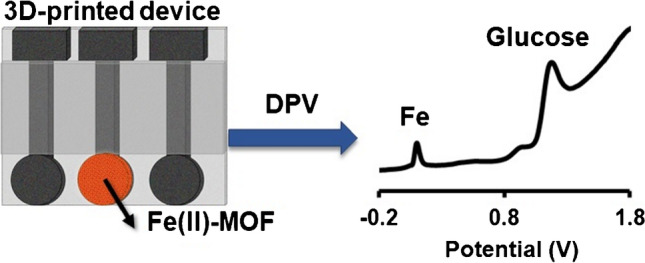

**Supplementary Information:**

The online version contains supplementary material available at 10.1007/s00604-023-05860-6.

## Introduction

Diabetes is one of the commonest and life-threatening diseases globally, causing serious problems to the nerves and blood vessels and leading to kidney failure, strokes, and blindness. Failure of the pancreas to produce insulin leads to elevated blood glucose (GLU) levels which is the main cause of diabetes [1, 2]. The periodical monitoring of GLU concentrations is typically carried out with commercially available electrochemical GLU self-testing devices. These methodologies are invasive as they demand a small volume of blood, which is mainly obtained by finger pricking, causing discomfort and pain after frequent use. Thus, there is ongoing effort to produce non-invasive GLU devices by assaying GLU in other biological samples, such as sweat, since this epidermal fluid contains GLU at levels that correlate well with blood [3, 4].

Electrochemical GLU devices can be classified into enzymatic and non-enzymatic [5, 6]. In enzymatic GLU sensors, a modified electrode with glucose oxidase (GOx) is used, but these devices suffer from poor stability (i.e., strong dependence on pH, temperature, and ionic strength) and are expensive [7, 8]. These limitations of enzymatic GLU sensors have prompted the development of new generations of non-enzymatic GLU sensors. In non-enzymatic electrochemical GLU sensors, metals, metal oxides, metal complexes, and recently metal–organic frameworks (MOFs) are utilized as electrode modifiers, which are capable of efficiently and directly catalyzing the oxidation of GLU [1, 2, 9–15].

The scientific field of MOFs has witnessed major achievements over the last years, and some of them have been introduced in the field of GLU sensing. MOFs are an advanced new class of hybrid porous crystalline materials constructed by assembling metal ions or metal clusters with multidentate rigid organic ligands via coordination bonds and they have numerous advantages over other porous materials, such as high surface area, tunable size of nanopores, and uniformly structured cavities [1, 2]. Some water-stable pristine MOFs can be used as artificial bio-mimic enzyme nanomaterials (defined as nanozymes) with specific catalytic and electrochemical redox activity [1, 2, 16–26]. Thus, the limitations of biological enzymes, in terms of low stability and high cost, are addressed by specific MOFs in the construction of electrochemical GLU biosensors. The mechanism of a MOF nanozyme in GLU sensing is based on the absorption of GLU on the surface and/or inside the pores of the MOF and the oxidation of GLU into gluconolactone caused by the redox activity of metal sites of the MOF, induced by the application and scanning of potential at the working electrode. Copper-, nickel-, and cobalt-based MOFs have been employed as electrode modifiers to construct enzymatic-free GLU electrochemical sensors [16–26]. For the construction of these MOF-based GLU electrochemical sensors, conventional glassy carbon and carbon-paste electrodes are used and the detection of GLU is carried out by amperometry in highly basic solutions (pH ≥ 12), while their applications are restricted to urine and serum samples (Table 1) [16–26]. Since these MOFs exhibit electrocatalytic activity for the oxidation of GLU only in alkaline media, this represents a very crucial issue in the detection of GLU, whose detection is preferable at physiologic and slight acidic pH, especially for the non-invasive monitoring in sweat.

**Table 1.** Comparison of electroanalytical performance of MOF-based nanozymatic GLU electrochemical sensors

MOF	Electrode	Electrochemical technique	Electrolyte	Sample	Studied concentration range (μmol L^-1^)	LOD (μmol L^-1^)	Ref.
Co-MOF	GCE*	Amperometry	NaOH 0.01 mol L^-1^	Urine	5–900	1.6	16
Mn/Co-MOF	GCE*	Amperometry	NaOH 0.1 mol L^-1^	Serum	50–900 and 1900–6900	1.31	17
Co/Ni/Fe-MOF	GCE*	Amperometry	NaOH 0.1 mol L^-1^	Serum	2–3000	1.13	18
Cu/Co-MOF	GCE*	Amperometry	NaOH 0.01 mol L^-1^	Serum, juice	5–400	1.6	19
Co-MOF	GCE*	Amperometry	NaOH 0.01 mol L^-1^	Serum, juice	10–1200	3.2	20
Cu-MOF	CPE**	Amperometry	NaOH 0.1 mol L^-1^	Serum	5–3910 and 3910–10,950	0.11	21
Cu-MOF	GCE*	Amperometry	NaOH 0.01 mol L^-1^	Urine	0.06–5000	0.0105	22
Ni-MOF	GCE*	Amperometry	NaOH 0.1 mol L^-1^	Serum	1–3000	1.0	23
Ni-MOF	GCE*	Amperometry	NaOH 0.1 mol L^-1^	Soft drinks	5–10,950	3.82	24
Ni/Mn-MOF	GCE*	Amperometry	NaOH 0.1 mol L^-1^	Serum	4.9–2200	0.87	25
Ni-MOF	GCE*	Amperometry	NaOH 0.1 mol L^-1^	Serum	1–500	0.25	26
Fe(II)-MOF	3D printed (CΒ/PLA)	DPV	PB 0.1 mol L^-1^ (pH 4)	Artificial sweat	100–600	17.6	This work

*GCE is glassy carbon electrode

**CP is carbon paste electrode

**Fig. 1:**
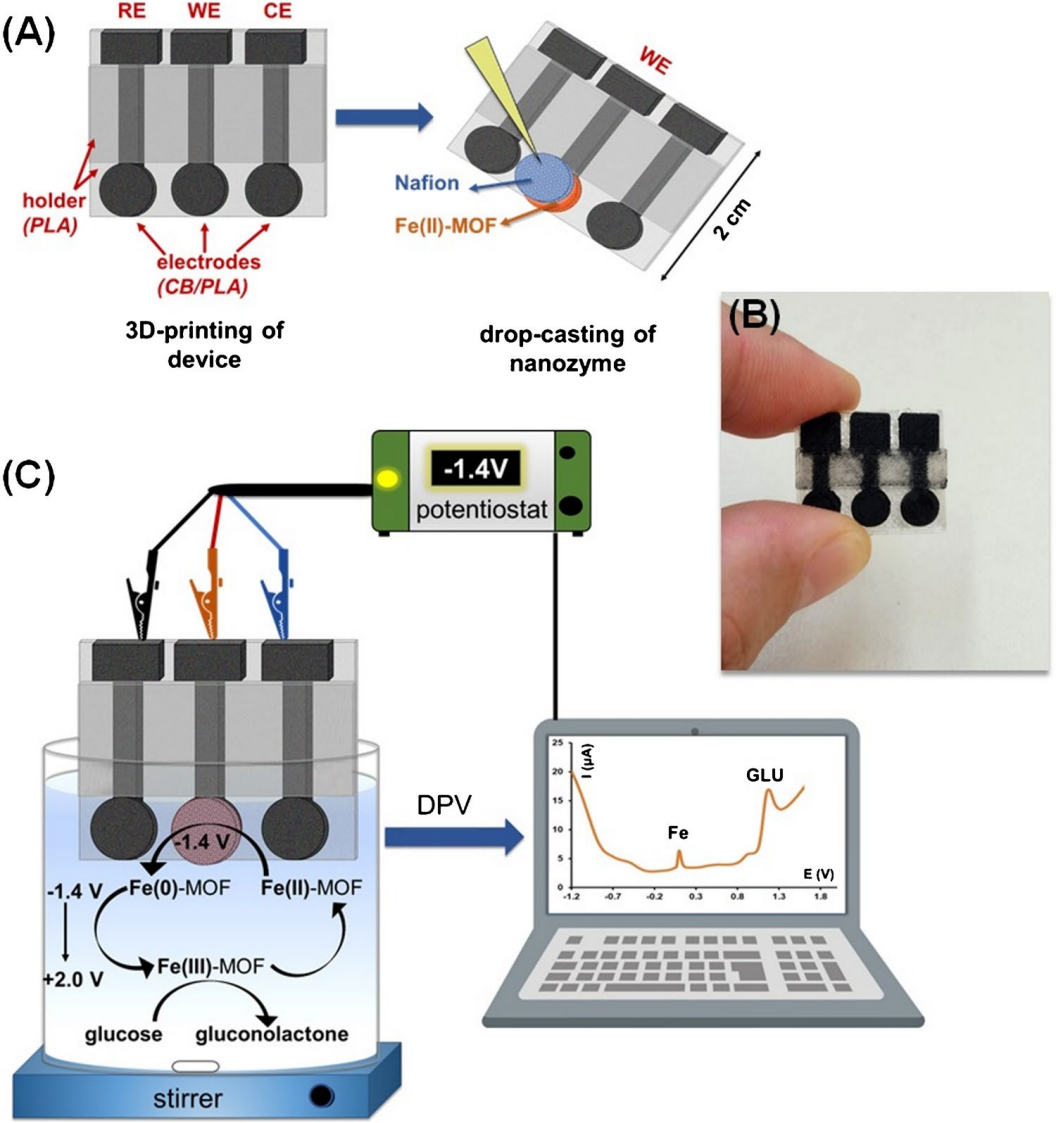
**A** Schematic illustration of the fabrication procedure of the GLU device by 3D printing using PLA and CB/PLA filaments and drop-casting of the Fe(II)-MOF nanozyme and Nafion film. **B** Photograph of the 3D-printed device. **C** Schematic illustration of the mechanism of the voltammetric detection of GLU at the MOF/3D-printed device

In this work, we have synthesized a water-insoluble and non-toxic Fe(II)-MOF (namely, {[Fe_2_(H_2_L^1^)_x_(H_2_L^2^)_y_(H_2_O)_2_]Cl_y_}_n_ (*x* ≈ 0.4, *y* = 1 − *x*), where H_6_L^1^ = 4,4′-[1,4-phenylenebis-(carbonylimino)]bis(2-hydroxybenzoic acid) and H_4_L^2^ = 4,4′-[1,4-phenylenebis-(carbonylimino)]bis(benzoic acid) (Figure S1-S3)) which served as a nanozyme for the differential pulse voltametric (DPV) GLU determination in artificial sweat. A 3D-printed device composed of 3 electrodes (printed by carbon black-polylactic acid filament (CB/PLA)) and a holder (printed by PLA filament) is printed in a single-step; and then, the Fe(II)-MOF is drop-casted on the surface of the working electrode, followed by its trapping with a Nafion film (Fig. 1). The key advantages, in terms of fabrication and operation, of the presented GLU device stem from the synergy of the MOF nanozyme and the application of the 3D printing technology. The Fe(II)-MOF can operate in acidic conditions (in contrast to other MOF-based GLU sensors which are applicable only in alkaline environments) and is suitable for enzyme-free GLU testing. The fabrication of the device by 3D-printing involves the use of a low-cost desktop-sized printer, cheap and non-toxic filaments, ease of operation, fast fabrication, and no waste, thus overcoming the weaknesses of existing manufacturing technologies applied in conventional electrochemical devices [27–31]. Additionally, the use of DPV as the electrochemical detection mode for GLU sensing provides higher selectivity than the commonly used amperometric detection, since DPV provides a fingerprint of the electrooactive components of the sample via the appearance of the characteristic voltametric peaks at different potentials.

## Materials and methods

### Chemicals and reagents

All chemicals and reagents were purchased from Sigma-Aldrich. A stock solution of 0.1 mol L^-1^ GLU was prepared in water, left for 24 h at room temperature for the isomer equilibration and then stored at 4 °C. The artificial sweat contained 3 mmol L^-1^ NH_4_Cl, 50 μmol L^-1^ MgCl_2_, 0.4 mmol L^-1^ CaCl_2_, 80 mmol L^-1^ NaCl, 8 mmol L^-1^ KCl, and 25 μmol L^−1^ uric acid; 22 mmol L^-1^ urea; and 5.5 mmol L^-1^ lactic acid [8, 11, 12]. The artificial sweat samples were analyzed without any treatment. For the preparation of the phosphate buffer (PB), appropriate quantities of Na_2_HPO_4_ and NaH_2_PO_4_ were mixed and the pH value was adjusted to 4 by addition of 1 mol L^-1^ of HCl.

### Synthesis of H_6_L^1^

#### 4,4′-[1,4-Phenylenebis-(carbonylimino)]bis(2-hydroxybenzoic acid)

In a 250-mL flat bottomed flask, 6.74 g (44 mmol) of 4-amino-2-hydroxy-benzoic acid is dissolved in 30 mL of N,N-dimethylacetamide, affording a clear brown solution (A). In a 100-mL beaker, 4.06 g (20 mmol) of terephthaloyl chloride is dissolved in 30 mL of N,N-dimethylacetamide, affording a clear pale-yellow solution (B). Solution B is poured slowly into solution A, and the new solution is stirred for 2 days at 50 °C. Finally, the contents of the final mixture are poured in a beaker containing 120 mL H_2_O (pH = 5) and the precipitation of the ligand starts rapidly, in the form of gray powder. The product is then filtered; washed with H_2_O (2 × 10 mL), EtOH (2 × 10 mL), and Et_2_O (2 × 10 mL); and dried in a vacuum oven (*P* = − 1 bar, *T* = 40 °C). Yield: 65.1%. ^1^H NMR (400 MHz, DMSO-*d*_6_) *δ* 11.40 (s, 2H), 10.62 (s, 2H), 8.10 (s, 3H), 7.79 (d, *J* = 8.7 Hz, 2H), 7.56 (s, 2H), 7.36 (dd, *J* = 8.7, 2.3 Hz, 2H).

### Synthesis of H_4_L^2^

#### 4,4′-[1,4-phenylenebis-(carbonylimino)]bis(benzoic acid)

In a 250-mL flat bottomed flask, 6.034 g (44 mmol) of 4-amino-benzoic acid is dissolved in 40 mL of N-methyl-2-pyrrolidone (A). In a 100-mL beaker, 4.06 g (20 mmol) of terephthaloyl chloride is dissolved in 40 mL of N-methyl-2-pyrrolidone, affording a clear pale-yellow solution (B). Solution B is poured slowly into solution A, and the new solution is stirred for 3 days at 50 °C. The ligand precipitates as a white powder and is filtered, washed with THF (2 × 10 mL) and dried in a vacuum oven (*P* = − 1 bar, *T* = 40 °C). Yield: 69.4%. ^1^H NMR (400 MHz, DMSO-*d*_6_) *δ* 12.77 (s, 2H), 10.69 (s, 2H), 8.13 (s, 4H), 7.96 (d, *J* = 2.7 Hz, 8H).

### Synthesis of Fe(II)-MOF

#### **{[Fe**_**2**_**(H**_**2**_**L**^**1**^**)**_**x**_**(H**_**2**_**L**^**2**^**)**_**y**_**(H**_**2**_**O)**_**2**_**]Cl**_**y**_**}**_**n**_**(*x*≈0.4,*y*= 1 −*x***)

Following a typical procedure [32, 33] with major modifications, in a 25-mL glass vial, 0.743 g of FeCl_2_∙4H_2_O (3.75 mmol), 0.175 g of H_6_L^1^ (0.4 mmol), and 0.242 g of H_4_L^2^ (0.6 mmol) are dissolved in 20 mL of N,N-dimethylformamide, affording a clear purple solution. After the addition of 3 mL of methanol, the final solution is transferred to a teflon lined autoclave vessel and placed in an oven for solvothermal reaction at 120 °C. After 3 days, the brown powder, which is formed, is filtered, washed with N,N-dimethylformamide (2 × 10 mL) and H_2_O (2 × 10 mL), and dried in a vacuum oven (*P* = − 1 bar, *T* = 40 °C).

For the activation of the product, it was immersed in 20 mL of H_2_O and stirred for 1 hour. It is then filtered; washed with H_2_O (2 × 10 mL), ethanol (2 × 10 mL), and diethyl ether (2 × 10 mL); and dried as mentioned before. Yield: 84.8%. IR (ATR): 3296 (m), 1652 (s), 1602 (s), 1572 (m), 1518 (s), 1421 (s), 1322 (s), 1268 (m), 1251 (m), 1181 (m), 1120 (w), 1017 (w), 994 (w), 898 (w), 863 (m), 854 (m), 778 (m), 698 (m), 655 (m), 502 (m), 413 (m).

[Fe_2_(H_2_L^1^)_x_(H_2_L^2^)_y_(H_2_O)_2_]Cl_y_}_n_ (*x* ≈ 0.6 and 0.8, *y* = 1 − x) were synthesized and treated as described above for the *x* ≈ 0.4, *y* = 1 − x product.

Fe-MOF-74 and Ni-MOF-74 were synthesized by following the reported procedure [33].

### Fabrication of the 3D-printed MOF-based GLU device

The 3D-printed device was designed with the Tinkercad software and was composed of 3 electrodes (working (WE), reference (RE), and counter (CE)) and a holder. The device was printed in a single-step using a dual extruder 3D printer (Creator Pro (Flashforge). The printing conditions were 60 °C for the platform, 200 °C for the two head dispensers, and 50 mm s^-1^printing speed. The filament used for the electrode printing was CB/PLA from Proto Pasta, while the filament used for the printing of the holder was transparent PLA from 3DEdge. Both filaments had a diameter of 1.75 mm. A quantity of 6 mg of Fe(II)-MOF powder was dispersed in 1-mL anhydrous ethanol under sonication for 20 min to form a uniform Fe(II)-MOF suspension containing 6 mg mL^-1^ (i.e., 0.6% w/v). Then, 10 μL of the 0.6% w/v Fe(II)-MOF ethanolic suspension was drop-casted on the cyclic surface of the WE and left 5 min for immobilization, followed by treatment with an air stream from a heat gun for 1 min. Then, 10 μL of 1% w/v ethanolic solution of Nafion was drop-casted on the 3D-printed MOF-modified surface of WE and left to dry for 5 min. Finally, the device was cured under an air stream from a heat gun for 1 min.

### Electrochemical measurements

The electrochemical measurements were carried out in a 10-mL electrochemical cell in ambient atmosphere and under stirring. The potentiostat was the EmStat3 (Palm Sens) and controlled by the PS Trace 4.2 software (Palm Sens). For the DPV measurements, a potential of − 1.4V for 120 s was applied to the WE followed by a DP scan (modulation amplitude, 50 mV; increment, 10 mV; pulse width, 75 ms; and pulse repeat time, 50 ms) and the DP voltammogram was recorded. The DPV peak of GLU appeared at about − 1.2 V. The cyclic voltammograms were obtained in 0.1 mol L^-1^ PB (pH 4) containing GLU at a scan rate of 50 mV s^-1^, after polarization of the WE − 1.4 V for 120 s. The connection of the three electrodes of the device to the potentiostat was accomplished using crocodile clips. All the potentials are referred with respect to the 3D-printed CB/PLA pseudo-reference electrode.

## Results and discussion

### Incorporation of deformities in the original Fe-MOF-74 analog and characterization

Fe(II)-MOF belongs in the family of M-MOF-74 (M = Mg, Co, Ni) analogs [32, 33] and constitutes a deformed version of the original M-MOF-74 structure. For the purpose of this work, the original Fe-MOF-74 and Ni-MOF-74 analogs (based only on the ligand H_6_L^1^, Figure S1) were also synthesized and tested, presenting almost no redox activity. Therefore, we reasoned that the increase of their electrochemical capabilities could be achieved through the introduction of some deformities in the already known structure using a second ligand (H_4_L^2^, Figure S1), which lacks one -OH group on each side [34]. This technique gave rise to the same network where there are three possible coordination modes for the Fe(II) ions, two of them containing open metal sites (Figure S2). To this end, we synthesized three deformed Fe(II)-MOFs, namely, {[Fe_2_(H_2_L^1^)_x_(H_2_L^2^)_y_(H_2_O)_2_]Cl_y_}_n_ (*x* ≈ 0.4, 0.6 and 0.8, *y* = 1 − *x*).

Since the Fe-MOF-74 and all deformed products were poorly crystalline, giving almost flat powder X-ray diffractions, we also synthesized and verified the structure of the Ni-MOF-74 analog using powder X-ray diffraction, in comparison to the corresponding one from the literature (Figure S4) [33]. Afterwards, we verified the structure of the Fe-MOF-74 by comparing its infrared (IR) spectrum with the IR spectrum of the original Ni-MOF-74 (Figure S5), as well as the IRs of all deformed Fe(II)-MOFs (Figure S6). Moreover, we were able to verify the degree of deformity in the deformed Fe(II)-MOFs by the lowering of the intensity of the peak at around 600 cm^-1^ which corresponds to the C-O stretching vibration of the aromatic hydroxyl groups (Figure S7), that represents the decrease of the -OH groups, as the deformity increases. Finally, the molecular formula of the product was verified by thermogravimetric analysis (Figure S8).

### Electrochemical characterization of the MOF-3D-printed GLU device

**Fig. 2:**
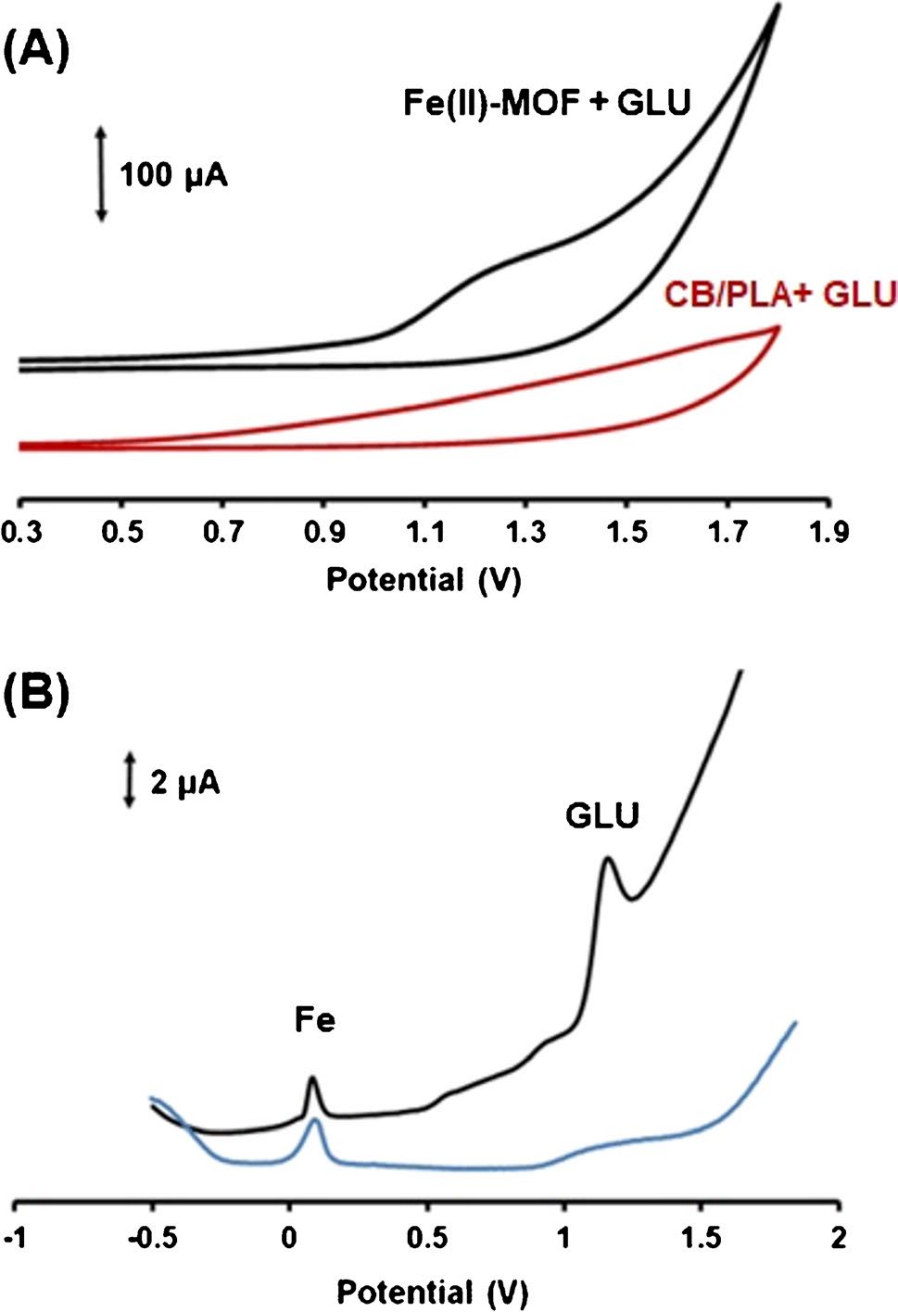
**A** CVs obtained with the bare 3D-printed CB/PLA electrode (red trace) and the 3D-printed CB/PLA electrode modified with the Fe(II)-MOF nanozyme (black trace) toward 1 mmol L^-1^ GLU in 0.1 mol L^-1^ PB (pH 4). **B** DP voltammograms obtained with the Fe(II)-MOF/3D sensor in the absence (blue trace) and presence (black trace) of 500 μmol L^-1^ GLU in 0.1 mol L^-1^ PB (pH 4). The 3D-printed CB/PLA sensor was modified with 0.6% w/v Fe(II)-MOF and a polarization potential at − 1.4 V for 120 s was applied

The 3D-printed sensor modified with Fe(II)-MOF and a bare 3D-printed CB/PLA electrode were characterized by cyclic voltammetry (CV), and the results are shown in Fig. 2A. In 0.1 mol L^-1^ PB (pH 4), the bare electrode did not reveal any oxidation peak in the presence of 1 mmol L^-1^ GLU, indicating that the bare CB/PLA electrode was inactive for electrooxidation of GLU, as expected. On the other hand, the 3D-printed CB/PLA sensor modified with the Fe(II)-MOF nanozyme presented an oxidation peak at about − 1.2 V in the presence of GLU, as glucose was oxidized to gluconolactone, demonstrating the ability of the Fe(ΙΙ)-MOF nanozyme to detect GLU in acidic conditions. Besides, Fig. 2B shows the DPV signals of the 3D-printed CB/PLA electrode modified with the Fe(II)-MOF in the absence and presence of 500 μmol L^-1^ GLU in 0.1 mol L^-1^ PB (pH 4). The MOF/3D device yielded a well-shaped and intense DPV oxidation peak for GLU in media with slight acidic pH. We can assume that the Fe(II)-MOF nanozyme absorbs GLU on its surface and oxidizes GLU to gluconolactone via the redox activity of Fe centers of the MOF to Fe(III), during the polarization of CB/PLA WE at − 1.4 V for at least 120 s and the following anodic potential scan. A possible mechanism of the electrocatalyzed oxidation of GLU to gluconolactone on the surface of the Fe(II)-MOF/3D-printed CB/PLA device can be described as follows (Fig. 1B) [12–15].

Possible mechanism of GLU oxidation induced by Fe(II)-MOF nanozyme:

A) *Redox of Fe-cluster of MOF:*

Reduction: Fe(II)_-MOF_ →Fe(0)_-MOF_ (polarization of WE at − 1.4 V for 120 s)

oxidation: Fe(0)_-MOF_ →Fe(III)_-MOF_ (DP scan from − 1.4 to + 2.0 V)

B) *Electrocatalyzed oxidation of GLU:*

2Fe(III)_-MOF_ + Glucose→2Fe(II)_-MOF_ + Gluconolactone +H_2_O

### Effect of Fe(II)-MOF loading, polarization time, and potential on GLU sensing

**Fig. 3:**
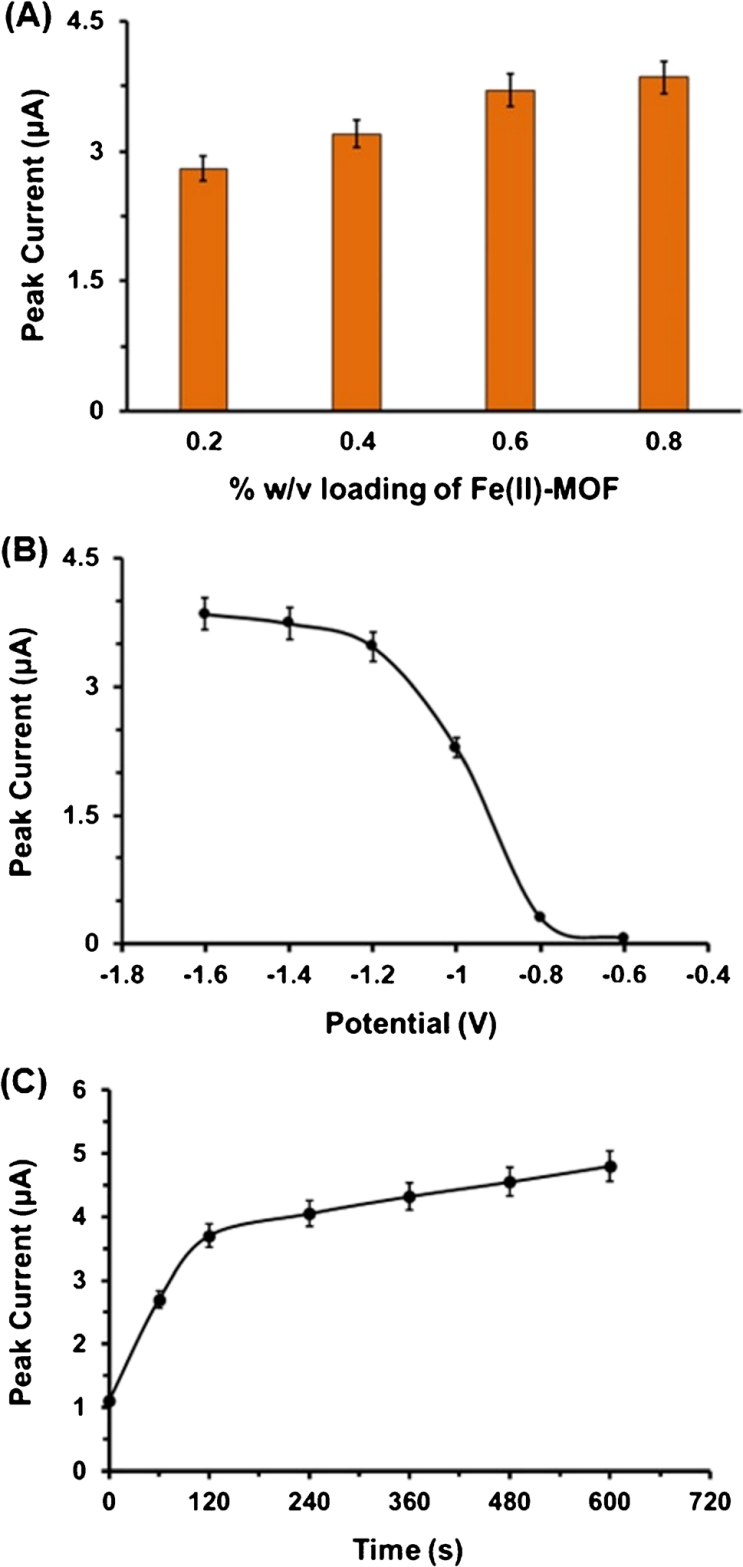
**A** Effect of the loading of the Fe(II)-MOF at the 3D-printed device on the DPV peak currents of 500 μmol L^-1^ GLU in 0.1 mol L^-1^ PB (pH 4). Polarization at − 1.4 V for 120 s. **B** Effect of the polarization potential on the DPV peak currents of 500 μmol L^-1^ GLU in 0.1 mol L^-1^ PB (pH 4) at MOF/3D-printed device under 120 s polarization time. **C** Effect of polarization time on the DPV peak current values of 500 μmol L^-1^ GLU in 0.1 mol L^-1^ PB (pH 4) at MOF/3D-printed device applying a polarization potential at − 1.4 V. Each bar and point is the mean value ± sd (*n* = 3)

The effect of the loading of the Fe(II)-MOF on surface of the 3D-printed WE, the polarization potential, and the polarization time were studied by recording the DP voltammograms of 500 μmol L^-1^ GLU in 0.1 mol L^-1^ PB (pH 4) (Fig. 3). The amount of the Fe(II)-MOF nanozyme on the 3D-printed WE influences the absorption of GLU and the number of the active Fe centers, while the polarization potential and the polarization time of the WE affect the redox activity of the Fe centers of the MOF, which induce the oxidation of GLU to gluconolactone. Four loading levels of the Fe(II)-MOF on the 3D-printed device in the range 0.2–0.8% w/v (as ethanolic suspensions) were assessed. As illustrated in Fig. 3A, the 0.6% w/v Fe(II)-MOF loading showed about 30% increase in voltammetric oxidation peak height of GLU over the 0.2% w/v loading and about 16% increase over the 0.4% w/v loading and practically similar sensitivity to the 0.8% w/v loading. Thus, a Fe(II)-MOF loading of 0.6% w/v was chosen for the experiments.

The effect of the applied value and duration of the polarization potential of MOF/3D-printed WE was tested in the range − 1.6 to − 0.6 V and from 60 to 600 s. As illustrated in Fig. 3B and C, the GLU oxidation signals were higher at more negative polarization potentials and at polarization times longer than 60 s. For the following experiments, a polarization potential of − 1.4 V for 120 s was chosen offering high sensitivity and short analysis time.

### Analytical evaluation, interferences, and application

**Fig. 4:**
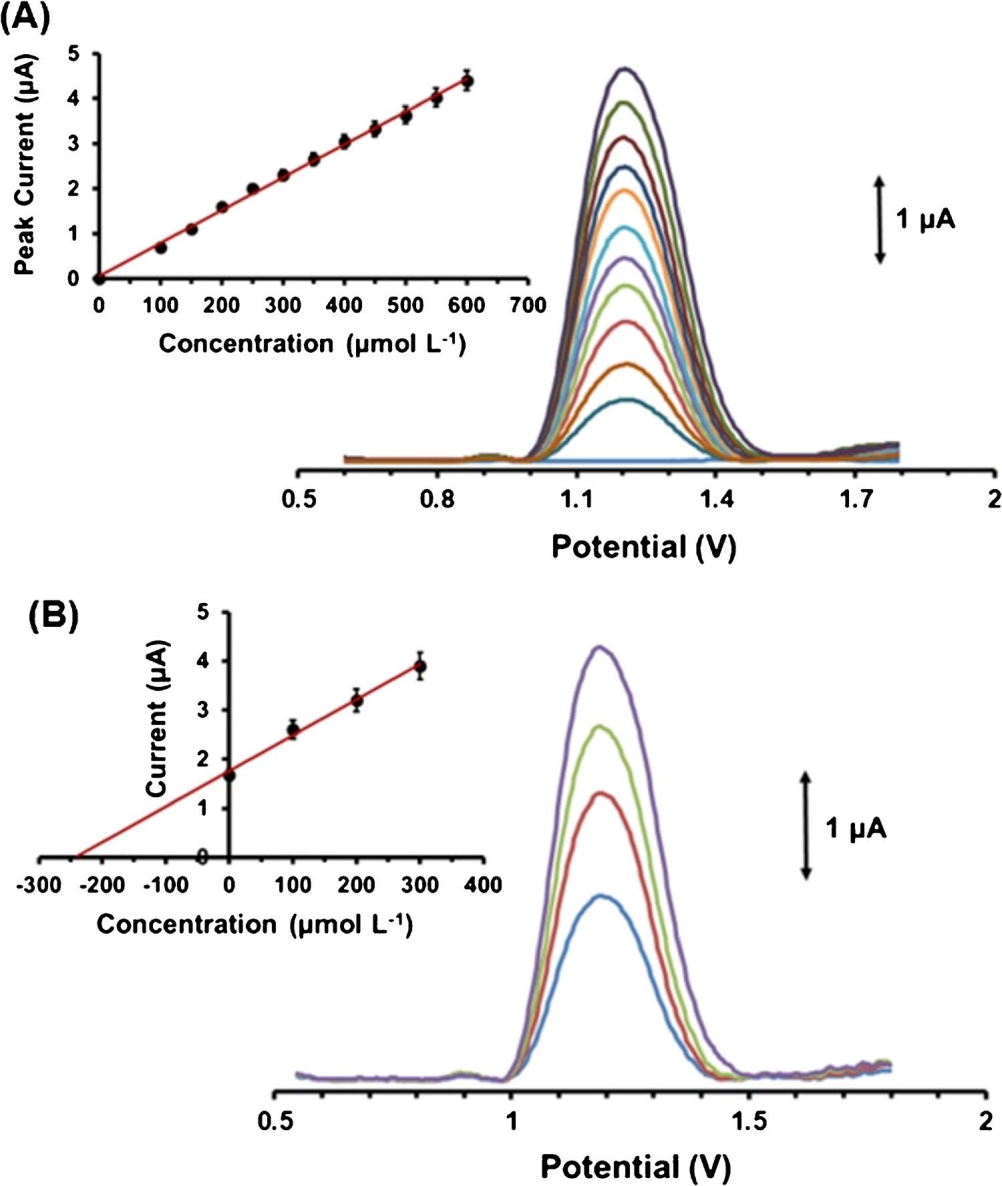
**A** Baseline-corrected DP voltammograms of GLU in the concentration range 0−600 μmol L^-1^ (from down to up: 0, 100, 150, 200, 250, 300, 350, 400, 450, 500, 550, and 600 μmol L^-1^ GLU) in 0.1 mol L^-1^ PB (pH 4) applying − 1.4 V for 120 s. **B** Baseline-corrected DP voltammograms and respective standard additions plot for the determination of GLU in an artificial sweat sample spiked with 250 μmol L^-1^ GLU. Each point in the calibration plots is the mean value ± sd (*n* = 3)

Figure 4A illustrates the DPV responses and the calibration plot of GLU at the MOF/3D-printed device. The calibration curve was linear in the concentration range 100–600 μmol L^-1^ GLU, with a correlation coefficient of 0.997; this linear concentration range falls within the physiological GLU levels secreted in human sweat [8, 11, 12]. The limit of detection (LOD) was 17.6 μmol L^-1^ and was calculated by the equation LOD = 3*s*_*y*_/*a*, where *s*_*y*_ is the standard deviation of the *y*-residuals of the calibration plot and *a* is the slope of the calibration. The within-device reproducibility (stated as the % relative standard deviation (%RSD) of eight repetitive DPV responses at the device) was 5.2% and the between-device reproducibility (expressed as the % RSD of DPV response at five different devices) was 8.8% (both at the 400 μmol L^-1^ GLU level). The sensing performances of the Fe(II)-MOF/3D-printed device and the other reported MOF-based electrochemical GLU sensors are compared in Table 1. The majority of the existing MOF-based GLU electrochemical sensors are based on conventional electrodes and the determination of GLU is accessed through amperometry in highly alkaline media (pH ≥ 12); therefore, they are unsuitable for GLU monitoring in the acidic epidermal sweat environment.

Selectivity is a core factor of the electrochemical sweat biosensors, since co-existing oxidizable biomarkers may affect GLU sensing. Such typical sweat metabolites are urea, lactic acid, and uric acid [8, 11, 12]. To investigate the selectivity of the MOF/3D-printed device, a concentration of 220 mmol L^-1^ urea, 55 mmol L^-1^ lactic acid, and 250 μmol L^-1^ uric acid was added separately and combined in artificial sweat containing 400 μmol L^-1^ GLU and their effect on the DPV oxidation peak of GLU was examined (Figure S9). These sweat metabolites did not statistically affect the DPV response of GLU oxidation, indicating the satisfactory selectivity of MOF/3D-printed device in sweat GLU monitoring.

The applicability of the nanozyme MOF/3D-printed device to GLU monitoring was tested through recovery experiments. For this purpose, two artificial sweat samples containing 250 μmol L^-1^ (Fig. 4B) and 350 μmol L^-1^ GLU were analyzed. The standard addition method was used for the estimation of GLU concentration in the sweat samples and of the respective recovery values. The recoveries values were 96 and 102%, respectively, confirming the accuracy of the methodology utilizing the MOF/3D-printed device.

## Conclusions

In this work, we have synthesized a novel Fe(II)-MOF which serves as a nanozyme catalyzing GLU oxidation and integrated it in an all-3D-printed device for the voltammetric determination of GLU in artificial sweat. The measurements were conducted in the dynamic range required for glucose monitoring in human sweat and the device exhibited satisfactory electroanalytical performance. Since 3D printing technology allows the fabrication of wearable electrochemical sensors employing flexible filaments, the MOF/3D-printed device paves the way for on-skin GLU monitoring [8, 35]. However, every effort to develop wearable sweat-based GLU devices should overcome the inherent inaccessibility of natural sweat and take account of the correlation uncertainty between the sweat and blood GLU levels [36–39].

## Supplementary information

ESM 1
