# Supplementary material for: 3D-printed electrochemical glucose device with integrated Fe(II)-MOF nanozyme
Source: Mikrochim Acta. 2023 Jun 24;190(7):274. doi: 10.1007/s00604-023-05860-6 (PMC10290614; doi:10.1007/s00604-023-05860-6)
Supplement: Supplementary file 1 — ESM 1 [file 604_2023_5860_MOESM1_ESM.doc]

**Supplementary Material**

**3D-printed electrochemical glucose device with integrated Fe(II)-MOF nanozyme**

Eleni Koukouviti ^1^, Alexios K. Plessas^2^, Varvara Pagkali^1^, Anastasios Economou^1^, Giannis S. Papaefstathiou^2^, Christos Kokkinos^1^*

*^1^Laboratory of Analytical Chemistry, Department of Chemistry, National and Kapodistrian University of Athens, 15771 Athens, Greece*

*^2^Laboratory of Inorganic Chemistry, Department of Chemistry, National and Kapodistrian University of Athens, 15771 Athens, Greece*

*E-mail:* [*christok@chem.uoa.gr*](mailto:christok@chem.uoa.gr)

## Structure analysis


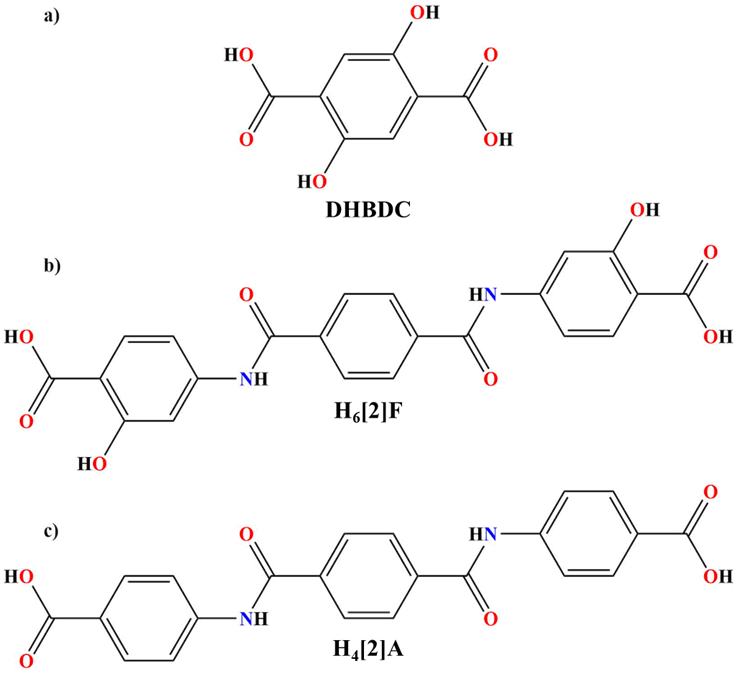


**Figure S1.** Representation of a) the classic ligand of MOF-74, b) the longer ligand bearing the same functional groups and c) the secondary ligand which lacks the hydroxyl groups.


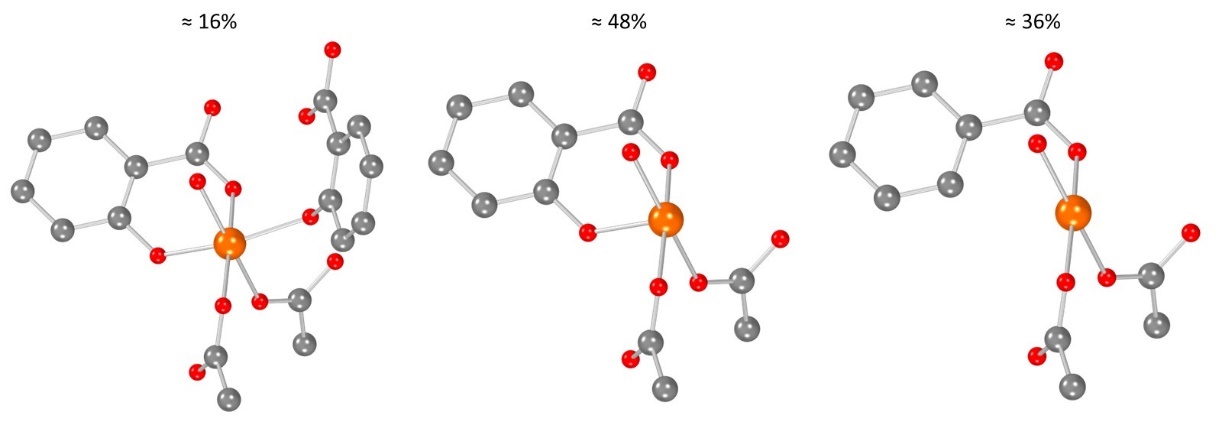


**Figure S2.** Representation of the three possible coordination modes of Fe(II) ions in Fe(II)-MOF.

Fe(II)-MOF’s structure is built by one dimensional chains (rods), formed by the carboxylic and hydroxy groups of the ligand bridging Fe(II) cations. Those chains are also bridged together sideways giving rise to a 3D network with hexagonal channels (Figure S3)


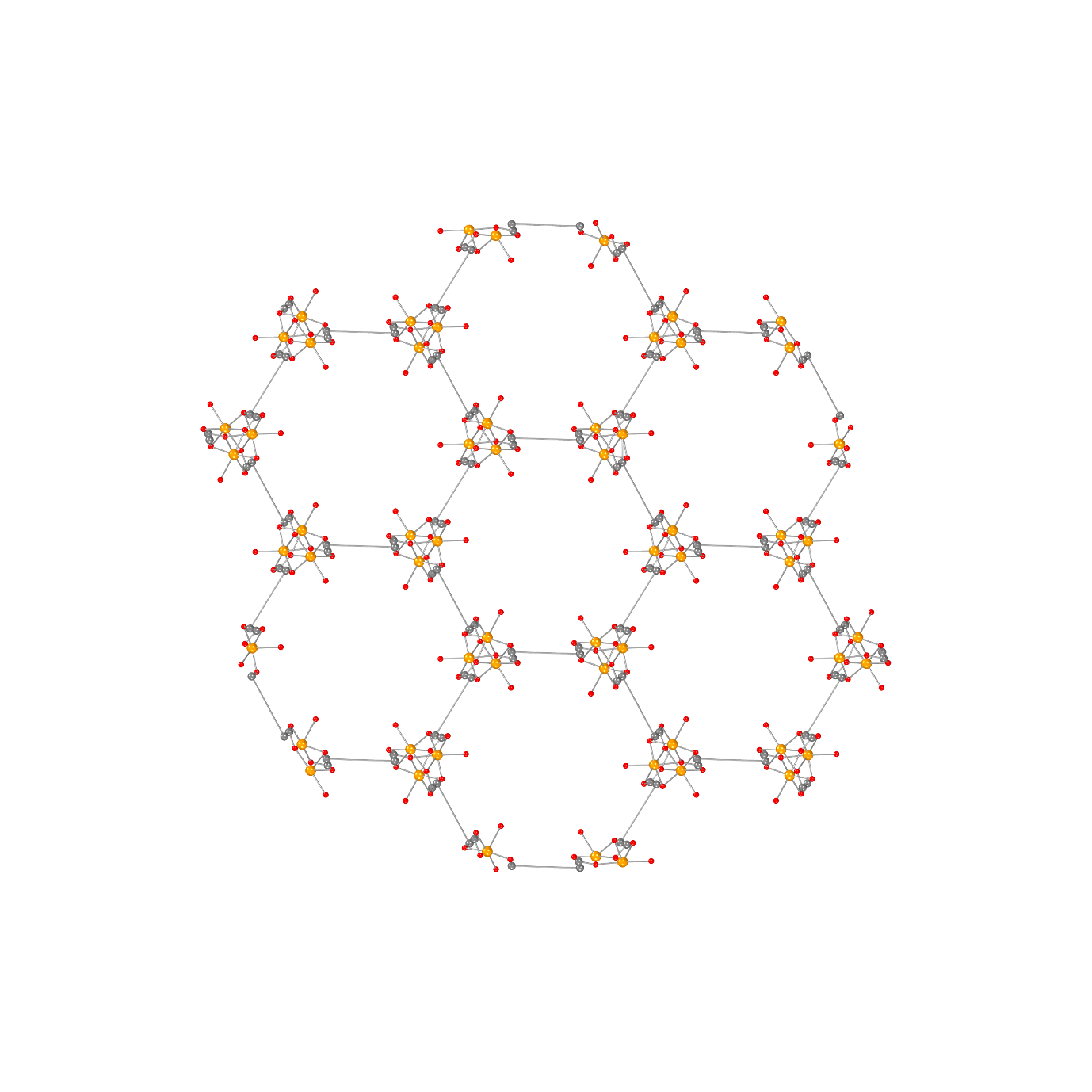


**Figure S3.** View of the 3D network of Fe(II)-MOF down to crystallographic axis c. Ligands are simplified as a solid lines (40% H_6_L^1^, 60% H_4_L^2^), connecting the carboxy and hydroxy groups on each side. Colour code: Fe=orange, O=red, C=grey, H=omitted for clarity.

## Powder X-Ray Diffraction


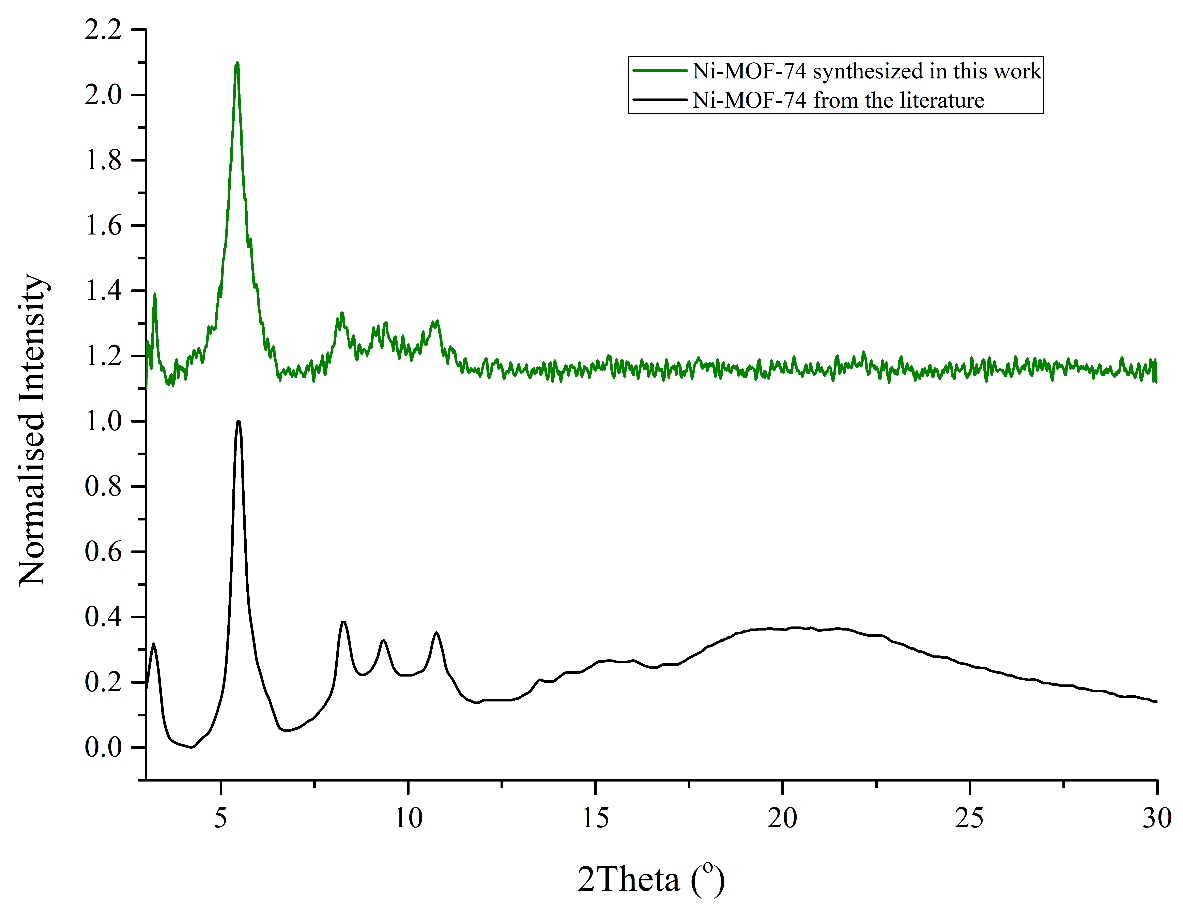


**Figure S4.** Powder X-Ray diffraction graph of the Ni-MOF-74 analogue compared to the corresponding one from the literature [1].

## Infrared Spectroscopy


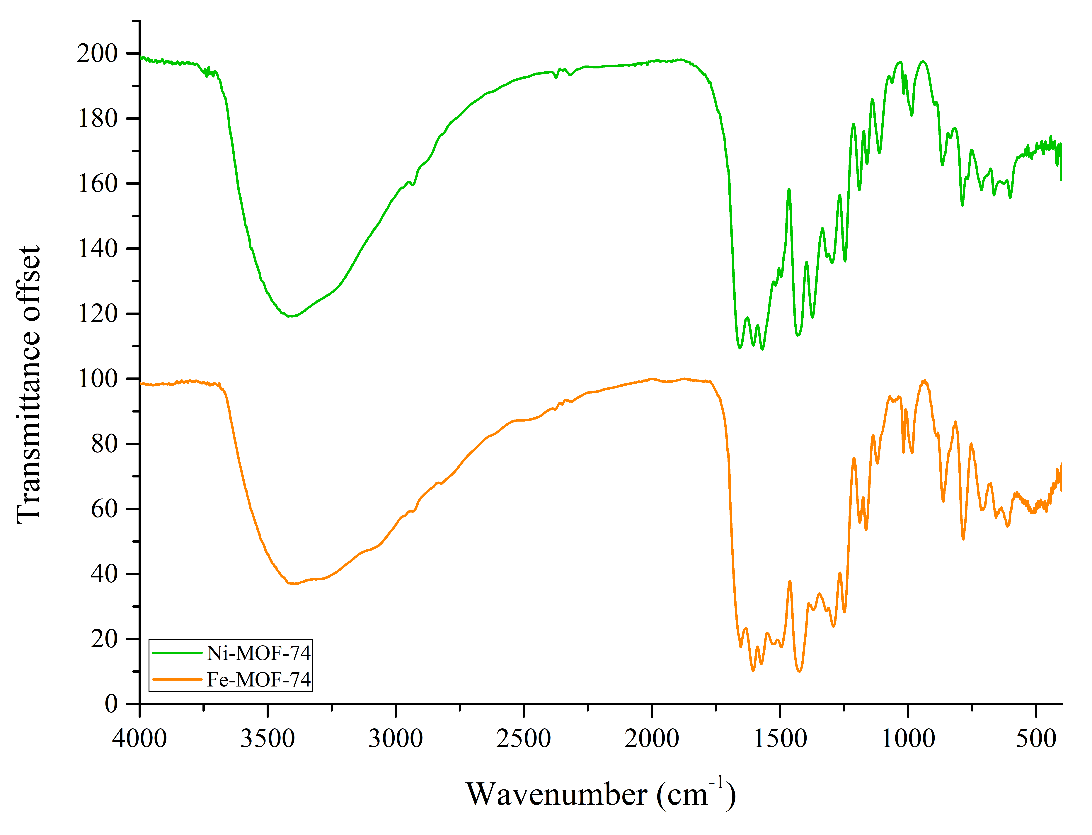


**Figure S5.** Infrared spectra (stacked) of the M-MOF-74 analogues (M = Ni, Fe).


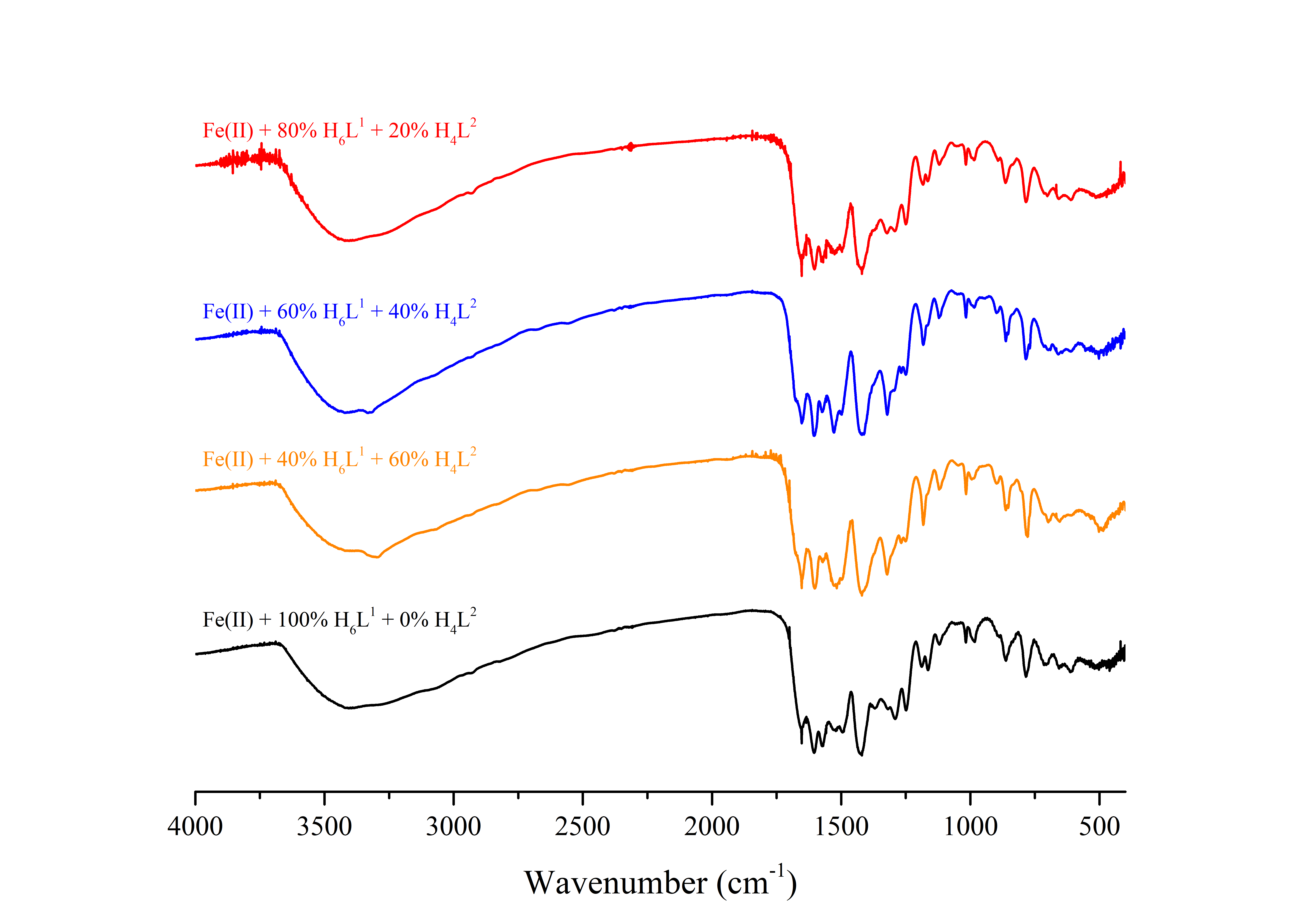


**Figure S6.** Infrared spectra (stacked) of the Fe-MOFs with different grades of deformity.


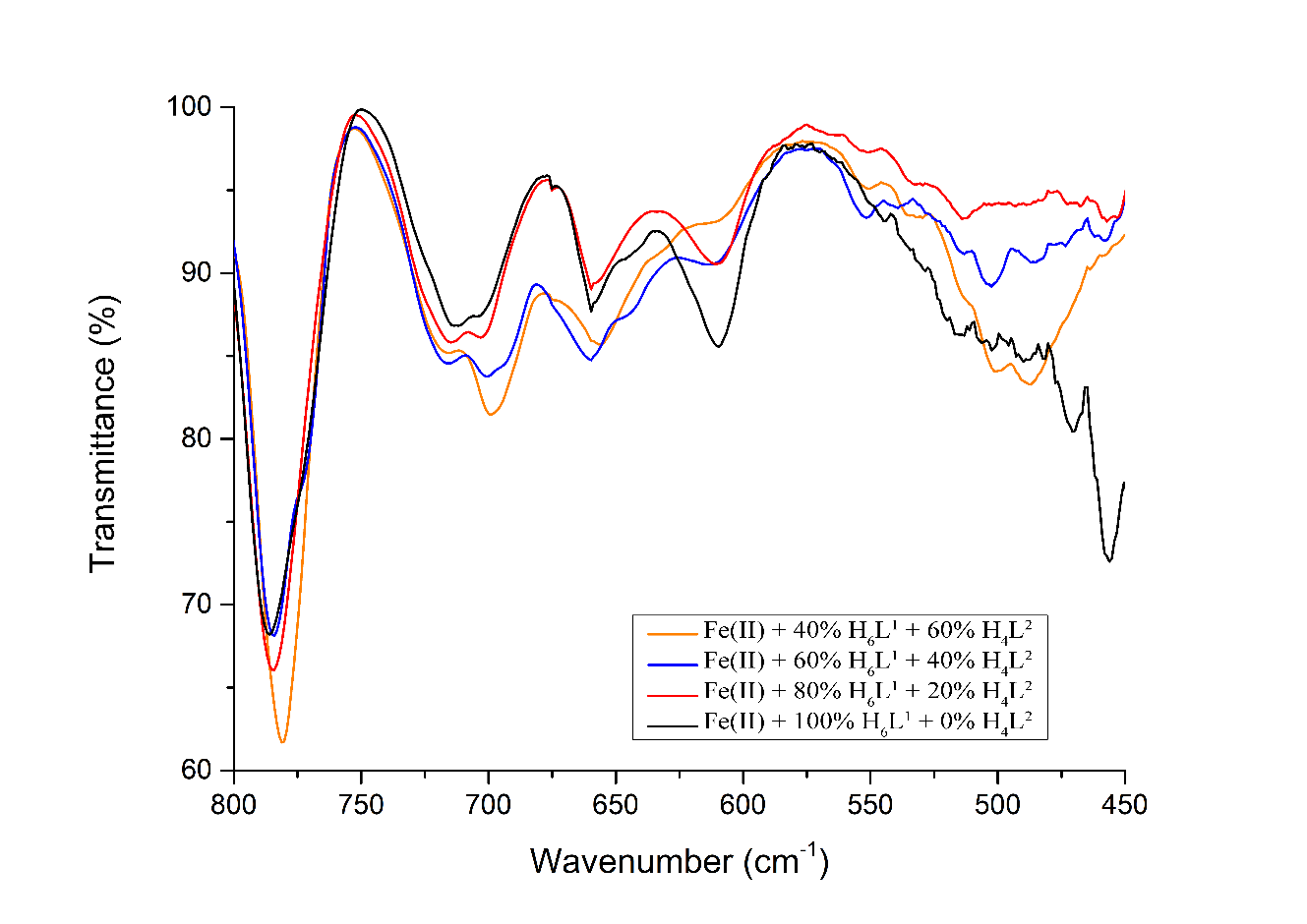


**Figure S7.** Infrared spectra (overlay) of the Fe-MOFs with different grades of deformity, zoomed in the region 800-450 cm^-1^.

**Thermogravimetric analysis**

From the thermograph below we can observe the loss of the 2 coordinated H_2_O molecules up to 148 ^o^C. Up to 200 ^o^C (melting point of the 3D printer’s filament) the product remains stable and starts to degrade afterwards, while the steps A-C cannot be assigned to a specific moiety loss. However the sum of the weight loss of those steps is in very good correspondence with the loss of 0.6 Cl^-^, 0.4 H_2_L^1^ and 0.6 H_2_L^2^ moieties. The mass of the final residue corresponds to FeCO_3_.


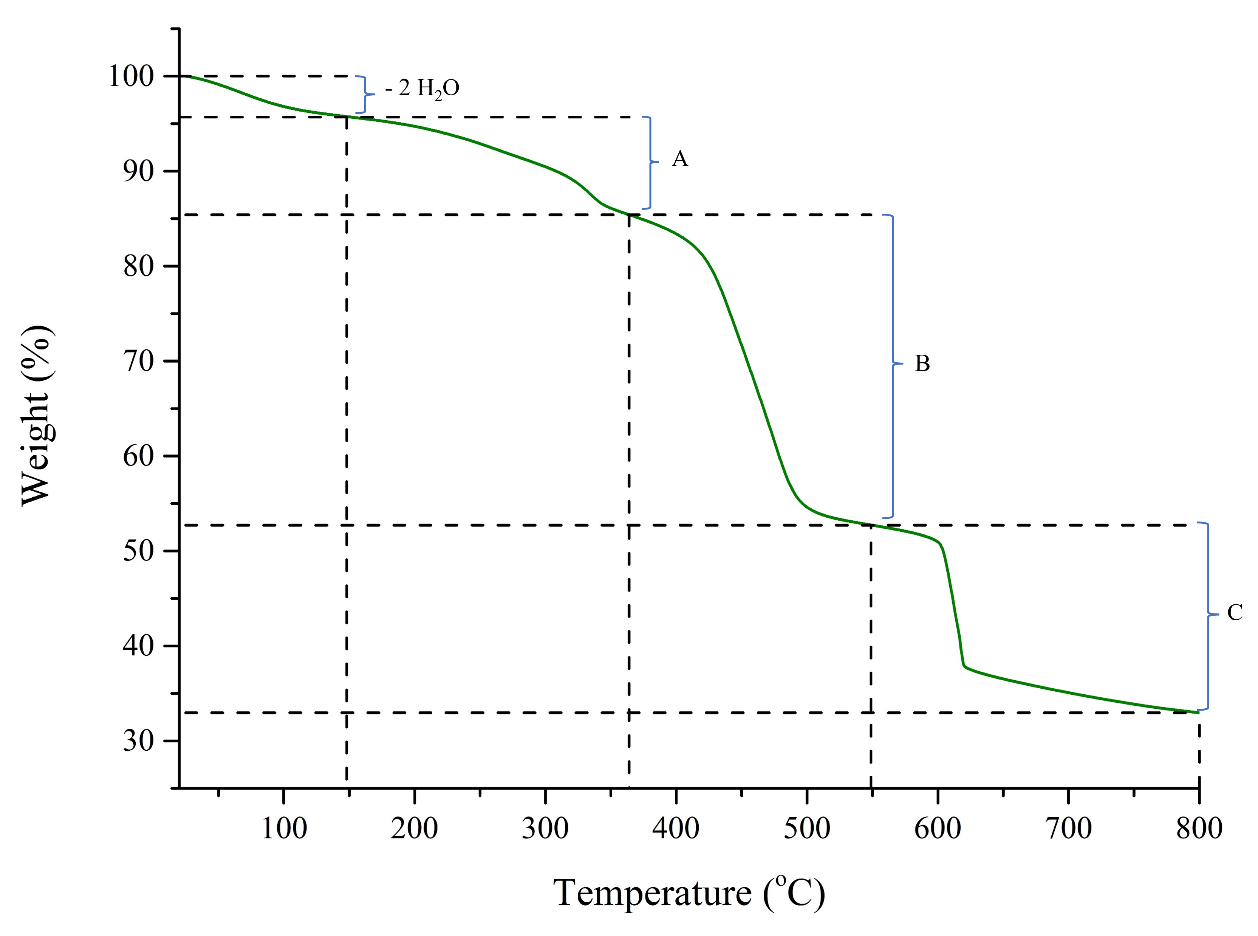


**Figure S8.** Thermogravimetric analysis graph for the Fe(II)-MOF in the range 25 – 800 ^o^C under nitrogen atmosphere.

**Interference study**


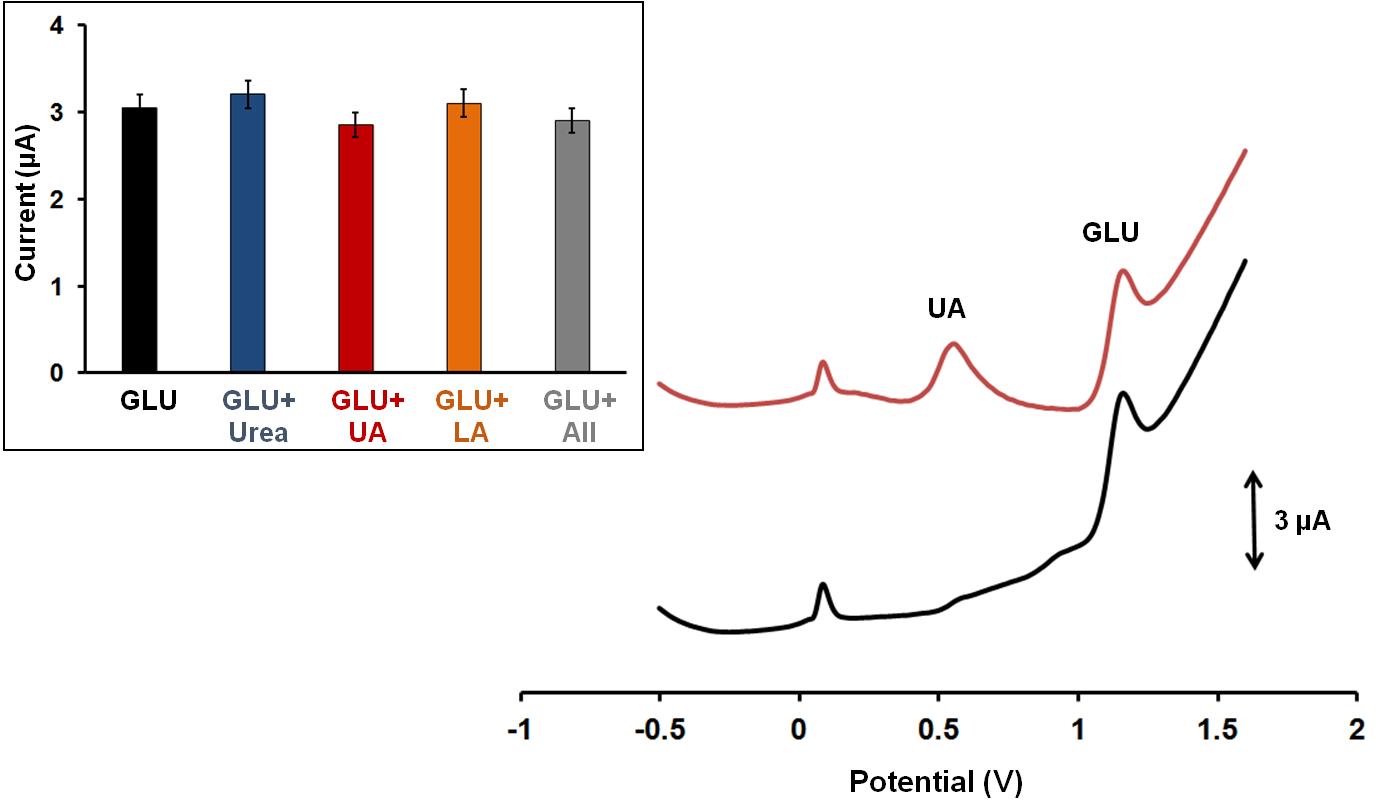


***Figure S9.****The black trace is the DPV response of 400 µmol L^-1^ glucose (GLU) at the nanozyme Fe(II)-MOF /3D printed device in artificial sweat (AS) and the red trace is DPV response of 400 µmol L^-1^ GLU in AS containing 250 µmol L^-1^ uric acid (UA), 220 mmol L^-1^ urea and 55 mmol L^-1^ lactic acid (LA). Inset the effect of UA, urea and LA on the DPV peak height of 400 µmol L^-1^ GLU, where: (black bar) 400 µmol L^-1^ GLU in AS; (blue bar) 400 µmol L^-1^ GLU + 220 mmol L^-1^ urea in AS; (red bar) 400 µmol L^-1^ GLU+ 250 µmol L^-1^ UA in AS; (orange bar) 400 µmol L^-1^ GLU + 55 mmol L^-1^ (LA) in AS; (grey bar) 400 µmol L^-1^ GLU + 220 mmol L^-1^ urea + 250 µmol L^-1^ UA + 55 mmol L^-1^LA in AS. Each bar is the mean value ± sd (n=3)*

# References

1] Nguyen B.T, Nguyen H.L, Nguyen T.C, Cordova K.E, Furukawa H (2016) High methanol uptake capacity in two new series of metal–organic frameworks: promising materials for adsorption-driven heat pump applications. Chemistry of Materials 28:6243-6249.
